# Extranodal T- and NK-cell lymphomas

**DOI:** 10.1007/s00428-022-03434-0

**Published:** 2022-11-07

**Authors:** Laurence de Leval, Andrew L. Feldman, Stefano Pileri, Shigeo Nakamura, Philippe Gaulard

**Affiliations:** 1 Institute of Pathology, Department of Laboratory Medicine and Pathology, Lausanne University Hospital and Lausanne University, 25 rue du Bugnon, CH- 1011 Lausanne, Switzerland; 2 Department of Laboratory Medicine and Pathology, Mayo Clinic, Rochester, MN USA; 3 Haematopathology Division, IRCCS, Istituto Europeo Di Oncologia, IEO, Milano, Italy; 4 Department of Pathology and Laboratory Medicine, Nagoya University Hospital, Nagoya, Japan; 5 Department of Pathology, University Hospital Henri Mondor, AP-HP, Créteil, France; 6 Inserm U955, Faculty of Medicine, IMRB, University of Paris-Est Créteil, Créteil, France

**Keywords:** T-cell lymphoma, Genomics, Diagnosis, Extranodal, Spleen, Gastrointestinal, Breast implant, International Consensus Classification

## Abstract

Non-cutaneous extranodal NK/T cell lymphoproliferations constitute a heterogenous group of rare neoplasms, occurring primarily in the gastro-intestinal tract, nasal area, spleen, and liver. Their nomenclature refers to their usual clinical presentation and predilection for specific anatomic sites—i.e. extranodal NK/T-cell lymphoma, nasal-type, hepatosplenic T-cell lymphoma, primary intestinal T-cell lymphomas, indolent lymphoproliferative disorders of the gastrointestinal tract, and breast implant-associated anaplastic large cell lymphoma. Extranodal tissues may also be involved by T-cell leukemias, or other entities usually presenting as nodal diseases. Primary extranodal entities range from indolent to highly aggressive diseases. Here, we will review the clinicopathologic features of the pertinent entities including the recent advances in their molecular and genetic characterization, with an emphasis on the changes introduced in the 2022 International Consensus Classification of lymphoid neoplasms, and highlight the diagnostic criteria helpful to sort out the distinction with potential mimickers.

## Introduction

Several neoplasms derived from mature T or NK cells characteristically present in specific extranodal sites [1, 6]. This paper is focused on non-cutaneous extranodal T/NK-cell lymphomas, including extranodal NK/T-cell lymphoma, nasal type (ENKTCL), primary intestinal T-cell lymphomas (ITCLs), indolent NK- and T-cell lymphoproliferative disorders (LPDs) of the gastrointestinal tract, hepatosplenic T-cell lymphoma (HSTCL), and breast implant-associated anaplastic large cell lymphoma (BIA-ALCL).

With the exception of ENKTCL, which is the most common type of peripheral T-cell lymphoma (PTCL) in Asia, most of these diseases are rare [73, 78]. Their identification and correct classification is of clinical importance as they differ in natural history, range from indolent to aggressive, and require different therapeutic approaches. In addition, they must be distinguished from NK- or T-cell leukemias or nodal lymphomas, which may involve extranodal sites at presentation or later in the disease.

We will review the clinicopathologic features of extranodal NK/T-cell neoplasms, including recent advances in their molecular and genetic characterization, based on the 2022 International Consensus Classification (ICC) of Mature Lymphoid Neoplasms (Table 1) [6]. We will highlight newly recognized entities and the diagnostic criteria helpful in distinguishing them from potential mimickers.

**Table 1:** Summary of 2022 ICC of non-cutaneous extranodal T and NK-cell neoplasms

2022 ICC designation	2017 WHO designation	Comments
Extranodal NK/T-cell lymphoma, nasal type	Extranodal NK/T-cell lymphoma, nasal type	No change in classification
Enteropathy-associated T-cell lymphoma	Enteropathy-associated T-cell lymphoma	No change in classification
Type II refractory celiac disease	Not listed	Type II refractory celiac disease was previously described under “enteropathy-associated T-cell lymphoma”, as its precursor lesion. Now defined as a distinct entity.
Monomorphic epitheliotropic intestinal T-cell lymphoma	Monomorphic epitheliotropic intestinal T-cell lymphoma	No change in classification
Intestinal T-cell lymphoma, NOS	Intestinal T-cell lymphoma, NOS	No change in classification
Indolent clonal T-cell LPD of the GI tract	Indolent T-cell LPD of the GI tract (provisional entity)	Upgraded to a definite entity with the introduction of “clonal” to emphasize the neoplastic nature of the disease and the need to establish clonality to distinguish from florid reactive lymphoid infiltrates
Indolent NK-cell LPD of the GI tract	Not listed	This disorder, formerly referred to as lymphomatoid gastropathy or NK-cell enteropathy, and briefly mentioned by analogy to similar T-cell-derived lymphoproliferations, is now defined as a distinct entity
Hepatosplenic T-cell lymphoma	Hepatosplenic T-cell lymphoma	No change in classification
Breast implant-associated ALCL	Breast implant-associated ALCL (provisional entity)	This entity was provisionally introduced in the 2017 WHO scheme and is now promoted to a definitive entity given its characteristic clinicopathologic features. No change in diagnostic criteria, use of the TNM staging system is recommended for disease evaluation

## Extranodal NK/T-cell lymphoma, nasal type

**Fig. 1:**
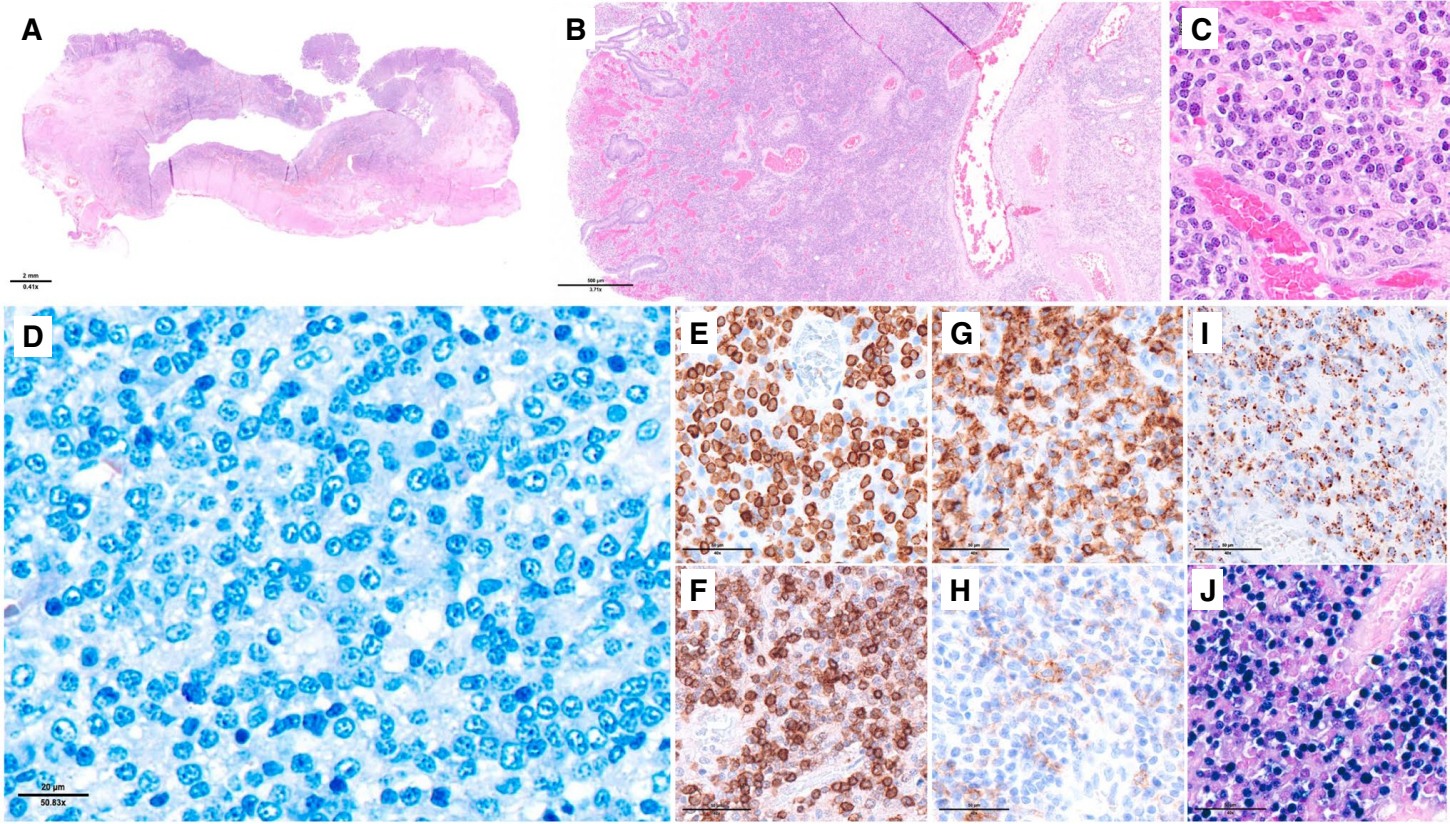
- Extranodal NK/T-cell lymphoma, nasal type involving the intestine. This case manifested as a post-transplant lymphoproliferative disorder in a patient with a kidney transplant. **A** Low magnification of the intestine showing an ulcerated infiltrative tumor disrupting the layers of the intestinal wall; **B** the ulcerated mucosa contained a dense lymphoid infiltrate obliterating the glands, prominent vessels and haemorrhage; **C** at higher magnification, the lymphoid infiltrate is monotonous; **D** a Giemsa stain highlights the nuclear features of the atypical medium-sized cells, showing round nuclear contours, slightly open chromatin and small nucleoli; **E**–**I** by immunohistochemistry the neoplastic cells are **E** CD3 + , **F** TCRbF1 + , **G** CD8 + , **H** focally and faintly CD56 + , and **I** granzyme B + ; **J** in situ hybridization with EBER probes is positive in most nuclei

The definition and diagnostic criteria of ENKTCL remain unchanged in the 2022 ICC. The name of the entity emphasizes its predilection for the nasal cavity and upper aerodigestive tract. Less commonly, ENKTCL presents in the skin, gastrointestinal tract (Fig. 1), testis, soft tissues, or other organs. These cases have a worse outcome than those with nasal presentation (34% versus 54% 5-year survival) [3]. Because ENKTCL is addressed in the companion paper on EBV-associated NK/T-cell lymphoproliferative disorders in this issue, it is only briefly summarized here (see Tables 2 and 3).

**Table 2:** Main clinicopathologic characteristics of non-cutaneous extranodal T and NK-cell lymphomas, and their differential diagnoses

2022 ICC entity	Clinicopathologic features	Phenotypic features	Differential diagnoses
Extranodal NK/T-cell lymphoma, nasal type	Ulcerated tumor in the nasal area, less commonly in skin, gastrointestinal tract or elsewhere; more common in Asia and Latin America Variable cytomorphology, angiocentricity, and necrosis	Positive: EBV (EBER +), CD2, CD3, CD56, TIA1, GzB and/or perforin Negative: CD4 Variable: CD30, p53, CD5, CD8 TCR: negative (most cases, NK cells) > TCRδ + > TCRβF1 +	Reactive inflammatory infiltrates with necrosis (non-specific, Wegener granulomatosis) Lymphomatoid granulomatosis EBV + mucosal CD30 + T-cell lymphoproliferative disorder Aggressive NK-cell leukemia Chronic active EBV disease Primary nodal EBV + T/NK-cell lymphoma
Enteropathy-associated T-cell lymphoma	Small intestinal tumor or ulcer in patients with preceding celiac disease or unknown celiac disease Pleomorphic tumor cells and polymorphic infiltrate	Positive: CD3, CD7, CD30, CD103, TIA1, GzB and/or perforin Negative: EBV, CD56, CD8 TCR: silent > TCRβF1 + > TCRδ +	Refractory celiac disease Monomorphic epitheliotropic intestinal T-cell lymphoma Anaplastic large cell lymphoma Intestinal T-cell lymphoma, NOS
Monomorphic epitheliotropic intestinal T-cell lymphoma	Small intestinal tumor Typically monomorphic medium-sized	Positive: CD8, CD56, CD2, CD3, CD7, CD103, TIA1 + GzB + / − perforin Negative: EBV, CD4, CD5, CD30, H3K36me3 Variable: CD20 and B-cell markers, MYC, p53 TCR: TCRδ + > TCRβF1 + > TCRβF1 − /TCRδ − or TCRβF1 + /TCRδ +	Enteropathy-associated T-cell lymphoma Indolent NK-cell or indolent clonal T-cell lymphoproliferative disorder of the gastrointestinal tract Extranodal NK/T-cell lymphoma, nasal type Extranodal marginal zone lymphoma of MALT type
Intestinal T-cell lymphoma, NOS	Presentation most commonly in the colon Morphology variable, usually pleomorphic	Positive: CD3 Negative: none Variable: EBV, CD30, CD56, TIA1 + GzB + / − perforin TCR: TCRβF1 + > TCRδ +	Enteropathy-associated T-cell lymphoma Monomorphic epitheliotropic intestinal T-cell lymphoma Peripheral T-cell lymphoma, NOS Anaplastic large cell lymphoma
Hepatosplenic T-cell lymphoma	Splenomegaly, cytopenias, hepatomegaly, frequent context of dysimmunity Medium sized cells, sinusoidal pattern	Positive: CD2, CD3, CD7, TIA1 Negative: EBV, CD4, CD57 Variable: CD8, CD56 TCR: TCRδ + > TCRβF1 +	T-cell large granular lymphocytic leukemia (T-LGLL) Aggressive NK-cell leukemia Intravascular lymphomas (bone marrow) Reactive expansions of γδ T cells
Breast implant-associated ALCL	Seroma + / − tumor lesion associated with breast implant Large anaplastic cells	Positive: CD30 (strong), CD4, CD45, EMA, Negative: ALK, EBV, CD5, CD7, B-cell markers Variable: CD3, CD8, TIA1, GzB, perforin, PD-L1 TCR: often silent	Periprosthetic reactive inflammatory effusions ALCL, ALK-negative Hodgkin lymphoma EBV + fibrin-associated large B-cell lymphoma

**Table 3:** Comparative features of NK/T-cell lymphomas and lymphoproliferative disorders (LPDs) involving the gastrointestinal (GI) tract

	Refractory celiac disease type II	Enteropathy-associated T-cell lymphoma	Monomorphic epitheliotropic intestinal T-cell lymphoma	Intestinal T-cell lymphoma, NOS	Indolent clonal T-cell LPD of the GI tract	Indolent NK-cell LPD of the GI tract	Intestinal extranodal NK/T-cell lymphoma, nasal type
Clinical presenting features	Persistance or recurrence of diarrhea and villous atrophy in < 1% of patients with celiac disease under gluten-free diet	Abdominal symptoms, diarrhea, obstruction, or perforation in patients with celiac disease or refractory celiac disease	Intestinal obstruction or perforation	Very rare, no predisposing factors known	Abdominal symptoms	Incidental discovery or abdominal symptoms	Bleeding or perforation
Localization	Small intestine	Small intestine > colon, stomach, often multifocal	Small intestine > colon	Colon > small intestine, stomach, extraintestinal spread	Small intestine and colon more commonly than upper GI tract	Stomach ("lymphomatoid gastropathy") more commonly than small intestine and colon	Small intestine or colon
Macroscopy/ endoscopy	Mucosal atrophy and/or ulcerative lesions (ulcerative jejunitis)	Ulcerations + / − strictures, infiltrative tumor; necrosis common	Infiltrative tumor; necrosis uncommon	Tumor	Mucosal ulcers or polyps, or normal-appearing mucosa	Erosions, ulcerations or normal-appearing mucosa	Infiltrative tumor; necrosis common
Histology	Villous atrophy, increased IEL, ulceration	Pleomorphic medium- to large-sized cells, angiocentrism and angioinvasion common	Monomorphic, small to medium-sized cells, epitheliotropism,	Pleomorphic medium to large cells	Non-destructive infiltrate, small lymphocytes without significant atypia	Usually non-destructive infiltrate, medium to large, atypical cells, no necrosis or IEL	Usually pleomorphic morphology, frequent angiocentrism, angioinvasion and necrosis
Immuno phenotype	sCD3 − CD3 + CD4 − CD5 − CD7 + CD8 − CD103 +	CD3 + CD4 − CD5 − CD7 + CD8 − CD103 +	CD3 + CD4 − CD5 − CD7 + CD8 + CD103 +	CD3 + CD4 CD8	CD3 + CD4 CD5 CD7 + / − CD8	CD3 + CD4 − CD5 − CD7 + CD8 −	cCD3 + CD4 − CD5 − CD7 + CD8 −
	CD30 −	CD30 +	CD30 −	CD30	CD30 −	CD30 −	CD30
	CD56 −	CD56 −	CD56 +	CD56	CD56 −	CD56 +	CD56 +
TCR expression	Negative	Negative > αβ > γδ	γδ > αβ or negative > double positive	Negative or αβ or γδ	αβ	Negative	Negative > αβ or γδ
Cytotoxic markers	Positive	Positive	Positive	Variable	Variable	Positive	Positive
EBV	Negative	Negative	Negative	Variable	Negative	Negative	Positive
Genes altered	*JAK1*, *STAT3*, *TNFAIP3*, *KMT2D*, *TET2*	*JAK1 STAT3*, *TNFAIP3*, *KMT2D*, *TET2*	*SETD2*, *STAT5B*, *JAK3*, *GNAI2*, *TP53*, *MYC*	*JAK1*, *JAK3*, *SETD2*, *STAT5B*, *TET2*	*STAT::JAK2* fusion, JAK/STAT pathway genes	*JAK3*, other miscellaneous genes	*BCOR*, *DDX3X*, *TP53*, *STAT3*

## T and NK-cell lymphomas and lymphoproliferative disorders of the gastrointestinal tract

The changes in the 2022 ICC regarding primary intestinal T/NK-cell neoplasms (Table 1) are (1) the definition of type II refractory celiac disease (RCD-II), formerly described as a precursor lesion to enteropathy-associated T-cell lymphoma (EATL), as a distinct disorder; (2) a change in the nomenclature for indolent T-cell LPD, now designated as indolent clonal T-cell LPD of the gastrointestinal tract; and (3) the introduction of a new entity, indolent NK-cell LPD of the gastrointestinal tract. Indolent clonal T-cell or NK-cell LPDs of the gastrointestinal tract are rare entities affecting middle-aged to older individuals. They are characterized by superficial involvement of the gastrointestinal tract, generally limited to the mucosa. They must be distinguished from ITCLs, which are usually aggressive diseases, and unlike lymphomas, these LPDs do not require (or respond to) chemotherapy.

ITCLs comprise two main entities, EATL and monomorphic epitheliotropic intestinal T-cell lymphoma (MEITL), plus ITCL, NOS, a default category for ITCLs with non-specific clinicopathologic characteristics. EATL and MEITL, formerly designated as variants of the same disease (EATL types I and II), were segregated as distinct entities in the 2017 WHO classification due to epidemiological, morphological, and immunophenotypic differences [1]. Recent molecular data reinforce this distinction [40, 44, 70]. The gastrointestinal tract can also be involved by any PTCL at presentation or later in the disease, especially ENKTCL. A comparative summary of gastrointestinal T- and NK-cell neoplasms is shown in Table 3.

### Type II refractory celiac disease (RCD-II)

RCD-II is a rare complication of celiac disease, a common immune-mediated enteropathy occurring in genetically susceptible individuals carrying HLA-DQ2 or HLA-DQ8, which is triggered and sustained by ingestion of gluten. RCD is defined as persistent GI symptoms and villous atrophy despite a strict gluten-free diet for at least 6–12 months, after other etiologies for non-responsiveness and malignancies have been excluded. Two types are recognized [54]. RCD-I is characterized by increased polyclonal intraepithelial lymphocytes (IELs) with a normal immunophenotype, whereas RCD-II represents a clonal proliferation of phenotypically aberrant IELs. RCD-II is associated with a poor prognosis and a 30–50% risk for transformation to overt EATL within 5 years [24]. RCD-II is therefore regarded as a precursor lesion to EATL or a cryptic intraepithelial T-cell lymphoma. Aberrant T lymphocytes in RCD-II are not strictly confined to the intestinal epithelium but may involve the lamina propria, other parts of the gastrointestinal tract, or extra-gastrointestinal locations, including the blood, bone marrow, and skin [4]. Patients with RCD-II often present with severe malnutrition, more pronounced than in RCD-I, due to chronic diarrhea and hypoalbuminemia related to large jejunal ulcers (ulcerative jejunitis), sometimes complicated by strictures [24, 32].

The prevalence of RCD-II is not known precisely, but is estimated to affect less than 1% of individuals with CD. Homozygosity for HLA-DQ2 and genetic polymorphisms at the 7p14.3 locus or in the *MYO9B* gene appear to increase the likelihood to progress from CD to RCD-II (or EATL) [24].

#### Morphology and immunophenotype

**Fig. 2:**
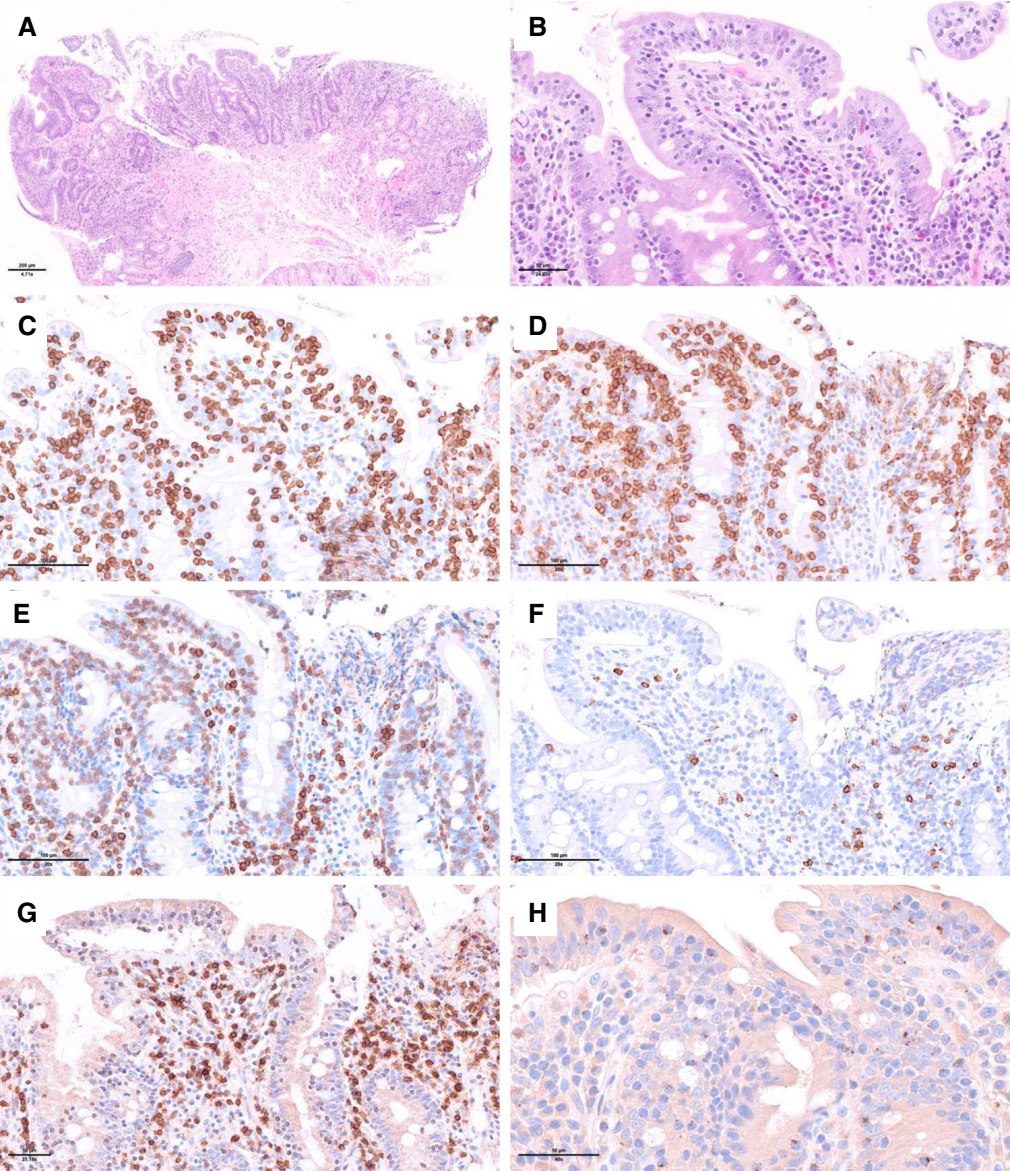
- Refractory celiac disease type II. **A** Duodenal biopsy showing subtotal villous atrophy; **B** at high magnification there is a marked increase in intraepithelial lymphocytes (IELs) devoid of significant atypia; **C**–**H** by immunohistochemistry **C** CD3 and **D** CD103 highlight the expanded IELs which are **E** weakly positive for CD5 (CD5dim), **F** negative for CD8, **G** show marked downregulation of TCRbetaF1 and **H** are partially positive for granzyme B. Molecular studies demonstrated monoclonal TCR gene rearrangements and pathogenic mutations in *STAT3*, *TET2*, and *KMT2D*

Small intestinal lesions in RCD-II show villous atrophy with expanded IELs, which are small with no or limited cytologic atypia. The histological features are similar to those of active CD or RCD-I, but mucosal ulceration is more frequent (Fig. 2).

The abnormal IELs in RCD-II have innate-like features with dual T- and NK-cell traits and are stimulated to expand by interleukin-15 (IL-15) overexpressed by the enterocytes in CD patients [19]. They are CD2 + , CD5 − , CD7 + , CD30 − , and CD56 − , and express αE integrin (CD103) and cytotoxic molecules (TIA1 + / − granzyme or perforin) [59]. They do not express surface CD3 (sCD3) but retain intracellular CD3 expression (cCD3), and usually lack surface expression of CD8 and T-cell receptor (TCR) molecules. By immunohistochemistry, a threshold of > 50% cCD3 + IELs showing loss of CD8 expression is used to indicate an aberrant immunophenotype [1]. Rare cases of RCD-II develop from TCRγδ IELs or from TCRαβ + IELs expressing CD8 or CD4 [34, 59] IELs in RCD-II also express the NK receptor NKp46 and one study found that detection of > 25 NKp46 + IELs per 100 epithelial cells discriminates RCD-II from CD and RCD-I [9]. Antibody–drug conjugates targeting NKp46 may represent a new therapeutic option for patients with RCD-II.

#### Molecular and genetic features

The abnormal IELs in RCD-II exhibit clonal *TRG* or *TRB* gene rearrangements. Trisomy of 1q is the only known recurrent chromosome abnormality [72]. Recent sequencing studies [13, 59] have identified genetic alterations in most cases, notably gain-of-function mutations in JAK-STAT pathway genes, including a *JAK1* p.G1097 hotspot mutations in about half of cases and frequent *STAT3*mutations, which appear to be the main drivers of CD-associated lymphomagenesis and confer hyper-responsiveness to IL-15 [59]. Other recurrent somatic events were observed in epigenetic regulators (*TET2*, *KMT2D*, *DDX3X*) and nuclear factor-κB (NFκB) pathway genes (*TNFAIP3*, *TNIP3*).

#### Differential diagnosis

The correct classification of RCD-I versus RCD-II is critical, since RCD-I follows a relatively benign course while RCD-II has a high risk of progression to EATL. Immunophenotypic characterization of the IELs and determination of clonality are key in making the distinction. While phenotypic characterization is most often performed by immunohistochemistry on endoscopic biopsies, flow cytometry is more sensitive, demonstrates the loss of surface CD3, and more precisely quantifies phenotypically aberrant IELs; flow cytometry is therefore regarded as the gold standard but not is not available in many centers [69]. NKp46 immunohistochemistry may be useful to identify RCD-II, but unfortunately, the antibody used by Cheminant et al. is not commercially available [9]. Demonstration of clonality is based on *TRB* and *TRG* gene rearrangement analyses. However, depending on the method used, a subset of RCD-II shows polyclonal patterns of TCR gene rearrangements, while monoclonal results are found in a proportion of uncomplicated CD or RCD-I [25]. Thus, integrative interpretation of the gene rearrangement findings with results of immunophenotyping and histopathology is necessary for accurate classification of RCD. The demonstration of somatic mutations in most RCD-II but not RCD-I suggests that high-throughput sequencing likely will aid the diagnostic work up of patients with RCD.

A clinical context of refractory CD may raise the differential diagnosis between EATL and RCD-II. Both consist of clonal and phenotypically aberrant lymphocytes, which in RCD-II are small cells typically confined to the epithelium, while EATL shows transformed large pleomorphic cells infiltrating the lamina propria and deeper layers of the intestine. Although EATL cells may invade the epithelium and cause confusion with RCD-II, they are usually larger and clearly atypical compared to the IELs in RCD-II, which are morphologically bland. The presence of atypical IELs with CD30 expression is rarely seen in RCD-II and is considered a sign of progression to EATL [41].

### Enteropathy-associated T-cell lymphoma

EATL is a lymphoma of intestinal intraepithelial T cells which occurs in individuals with CD and shows cellular pleomorphism and often an inflammatory component. The disease is more prevalent in Western populations. EATL was previously reported to be almost absent in Asians, but a recent epidemiological study conducted in South Korea, China, and other Asian countries indicates a prevalence close to that of MEITL [78]. The median age at presentation is over 60 years and most reports indicate a male predominance. EATL typically occurs in patients with CD or RCD, but up to half of patients have no previous history [22]. Homozygosity for HLA-DQ2 alleles and older age are risk factors [33]. EATL presents with gastrointestinal symptoms often culminating in intestinal perforation and peritonitis, or obstruction. EATL most commonly affects the small bowel and rarely the large intestine or stomach; lesions are frequently multifocal. Occasionally, EATL may present outside the gastrointestinal tract (e.g., skin, spleen, or CNS), especially cases evolving from RCD-II [34, 71]. The prognosis is poor, with a median survival of 7 months [48].

#### Morphology and immunophenotype

**Fig. 3:**
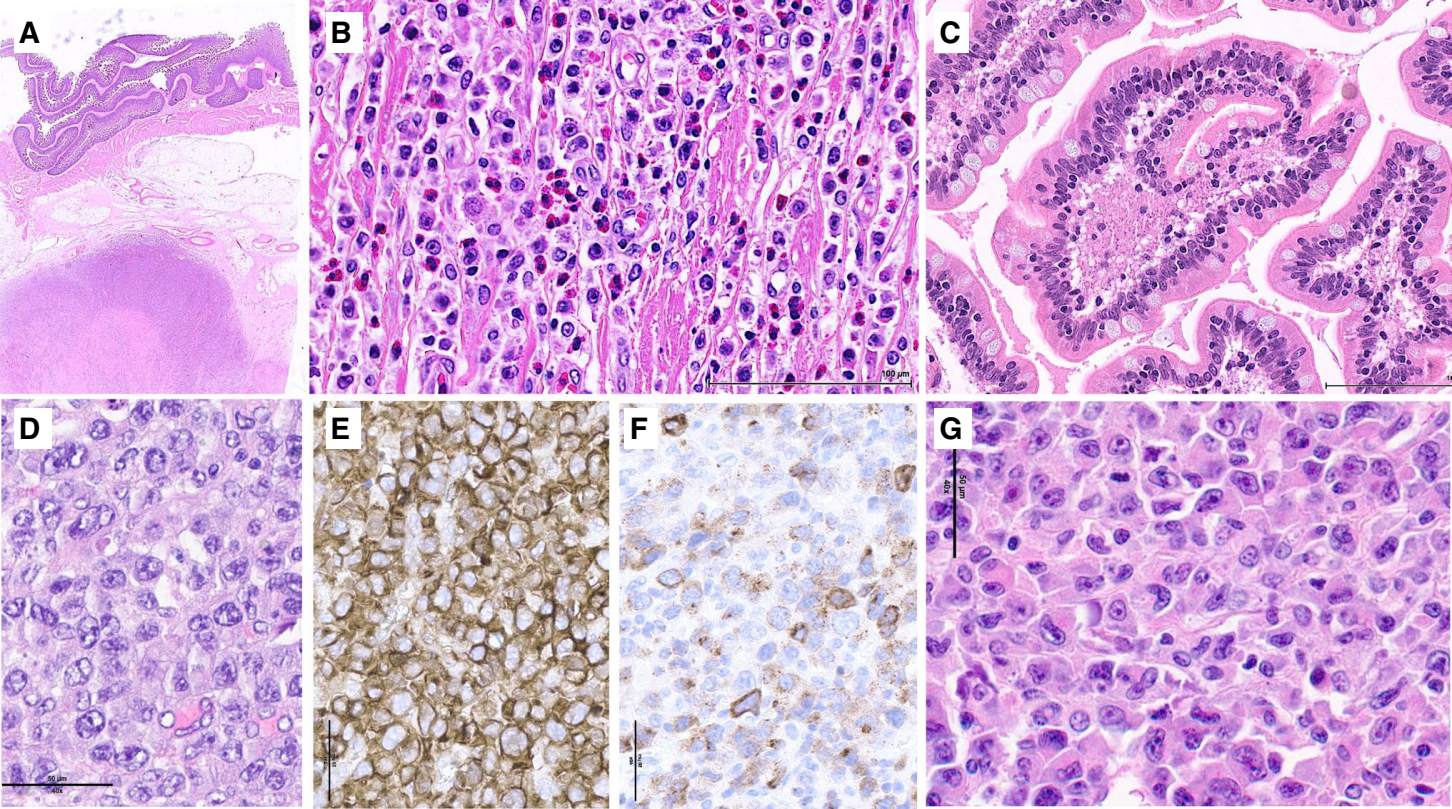
- Enteropathy-associated T-cell lymphoma. **A**–**C** This EATL consisted of **A** a large infiltrative mass with areas of necrosis and **B** comprised a polymorphic infiltrate rich in eosinophils and pleomorphic atypical lymphoid cells; distant mucosa **C** showed preserved villi but increased IELs. **D**–**F** This other EATL consisted of **D** large lymphoid cells with anaplastic features, and was **E** strongly CD30-positive, and **F** positive for perforin. **G** In this other case, the neoplastic lymphoid cells were admixed with many histiocytes

EATL manifests as ulcerative lesions with or without strictures, or tumor masses (Fig. 3). Mesenteric infiltration and lymph node involvement is common [22, 33]. The tumor can be monomorphic or pleomorphic, with a morphological spectrum ranging from medium to large cells with prominent nucleoli, to anaplastic-type or bizarre multinucleated cells [33]. Angiocentricity and angioinvasion, resulting in extensive necrosis, and a high mitotic rate are common. A polymorphic inflammatory infiltrate rich in eosinophils, plasma cells, and histiocytes is frequent, sometimes obscuring the lymphoma cells. Tumor cells may disseminate to distant intestinal epithelium or mucosa. Histopathologic features of CD may be seen outside the tumor.

EATL cells are usually CD2 + , CD3 + , CD4 − , CD5 − , CD7 + , CD8 − , and CD56 − , and express cytotoxic markers (TIA1, granzyme B and/or perforin) [55]. Up to 50% of de novo EATL express CD8 [33]. Most tumors displaying large cell morphology are CD30 + [5, 55]. They are negative for ALK and EBV. Most cases express CD103 [42] and are negative for TCR expression (“TCR-silent”); approximately 25% are TCRαβ + or express cytoplasmic TCRβ chains, and TCRγ chain expression has been rarely described [10, 33, 43, 44]. Overexpression of p53 is seen in many cases.

#### Molecular and genetic features

Clonal TCR gene rearrangements are detected in most cases. EATL demonstrates multiple chromosomal imbalances: 9q gains and nearly mutually exclusive losses at 16q12.1; gains of chromosome 7, 1q, and 5q; and losses involving 8p22-23.2, 16q21.1, 11q14.1-q14.2, and 9p21.2-p21.3 [17, 40]. The mutational landscape of EATL overlaps that of RCD-II and appears to be similar in cases developed from RCD-II and those occurring de novo [13]. Hence, EATL is characterized by highly recurrent activating mutations in the JAK/STAT pathway, most commonly in *JAK1* (at the p.c1097 hotspot) and *STAT3,* and rarely in *JAK3*, *STAT5B, TYK2,* or *SOCS1*. Other mutations occur in NFκB genes (*TNFAIP3*), genes involved in epigenetic regulation and gene expression (*KMT2D*, *BCOR*, *DDX3X*), mitogen-activated protein kinase (MAPK) pathway genes (*KRAS*, *NRAS*, *BRAF*), and *TP5*3 [13, 40, 46, 52].

#### Differential diagnosis

Establishing a diagnosis of EATL in patients with no history of CD may be difficult if enteropathic features are not seen outside of the tumor. If the pathologic features are suggestive or consistent with EATL, especially if supported by CD103 expression [42], the diagnosis should be suggested and serologic testing and HLA genotyping performed. In cases without CD, a diagnosis of intestinal T-cell lymphoma, NOS is more appropriate.

Diagnosing EATL outside the small intestine may be challenging; in patients with CD, any T-cell LPD, even outside the intestines, should be raise the diagnostic consideration of EATL or RCD [4].

EATL may be strongly CD30 + and resemble ALCL, with cytotoxic marker expression and loss of several T-cell antigens representing additional shared features. Primary intestinal ALK-negative ALCL is rare and this diagnosis should be considered only after thorough clinical investigation to exclude underlying CD. CD7 and CD103 may be helpful, as they are usually expressed in EATL and negative or downregulated in ALCL [42].

### Monomorphic epitheliotropic intestinal T cell lymphoma

MEITL is a rare primary intestinal T-cell lymphoma unrelated to CD, defined as a tumor composed of medium-sized cells with round nuclei and a rim of pale cytoplasm, typically showing striking infiltration of intestinal epithelium and lacking the inflammation and necrosis characteristic of EATL [1]. MEITL affects older adults. It occurs worldwide and is the main ITCL in Asia [16, 68]. A male predominance has been reported in several Asian series but not in most Western cohorts [23, 70].

Most patients present with acute symptoms, mainly related to intestinal perforation and/or obstruction by an infiltrative tumor mass. MEITL usually involves the small intestine, sometimes with concurrent colonic lesion(s). Presentation in the colon or stomach is rare. Dissemination to regional lymph nodes and distant organs may occur. The bone marrow is rarely involved. Prognosis is poor and the median overall survival is 7–15 months [23, 64, 68, 70].

#### Morphology and immunophenotype

**Fig. 4:**
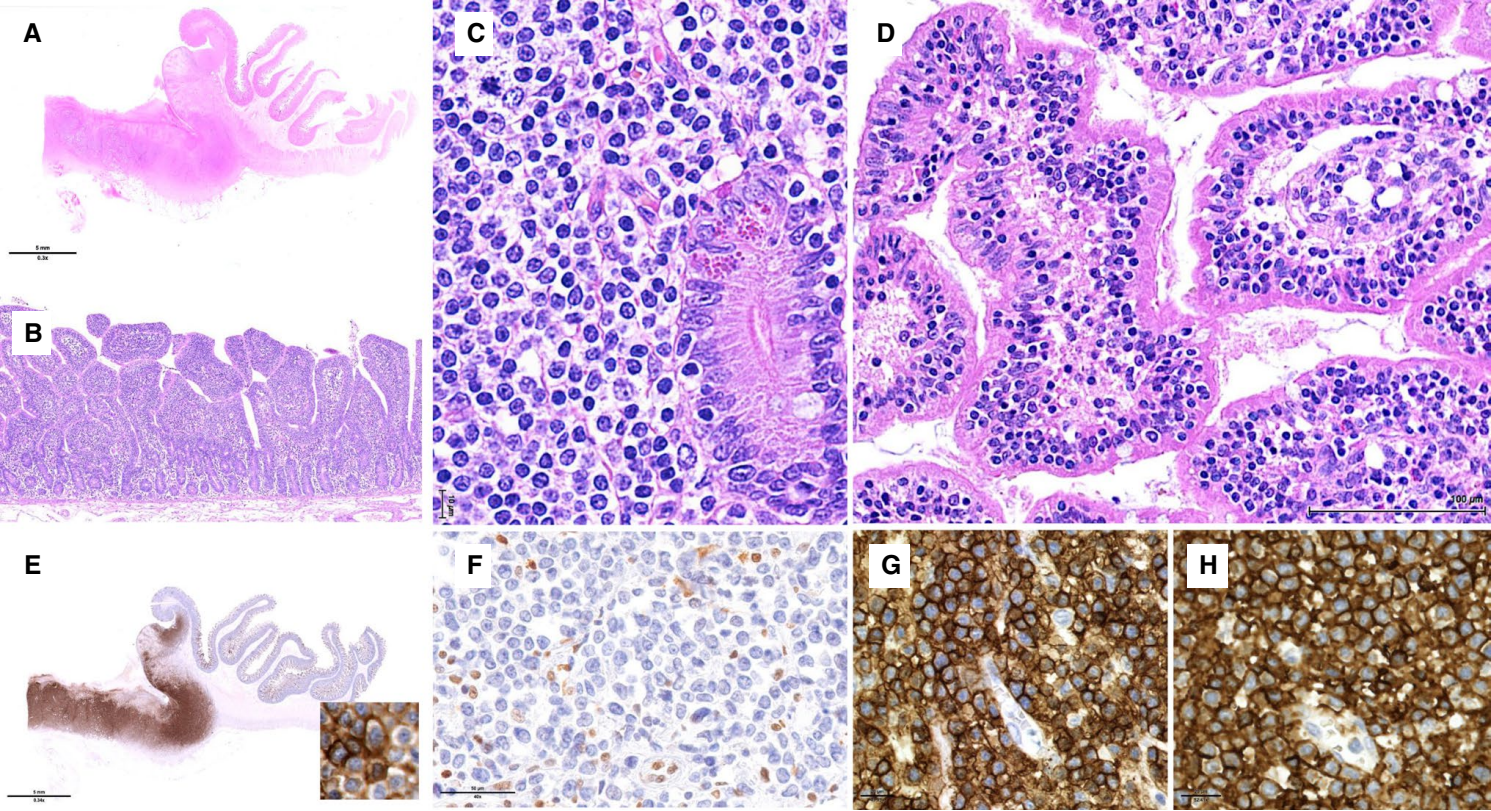
– Monomorphic epitheliotropic intestinal T-cell lymphoma. **A**–**B** Typical low-power appearance showing **A** a transmural ulcerated tumor with **B** lateral mucosal spread; **C** typical MEITL cytomorphology consisting of small to medium-sized cells with round nuclei and clear cytoplasm, **D** with intraepithelial spread to the adjacent mucosa; **E**–**H** usual MEITL immunophenotype characterized by **E** expression of CD103, strong and diffuse in this case, **F** lack of H3K26me3, and expression of **G** CD8 and **H** CD56

**Fig. 5:**
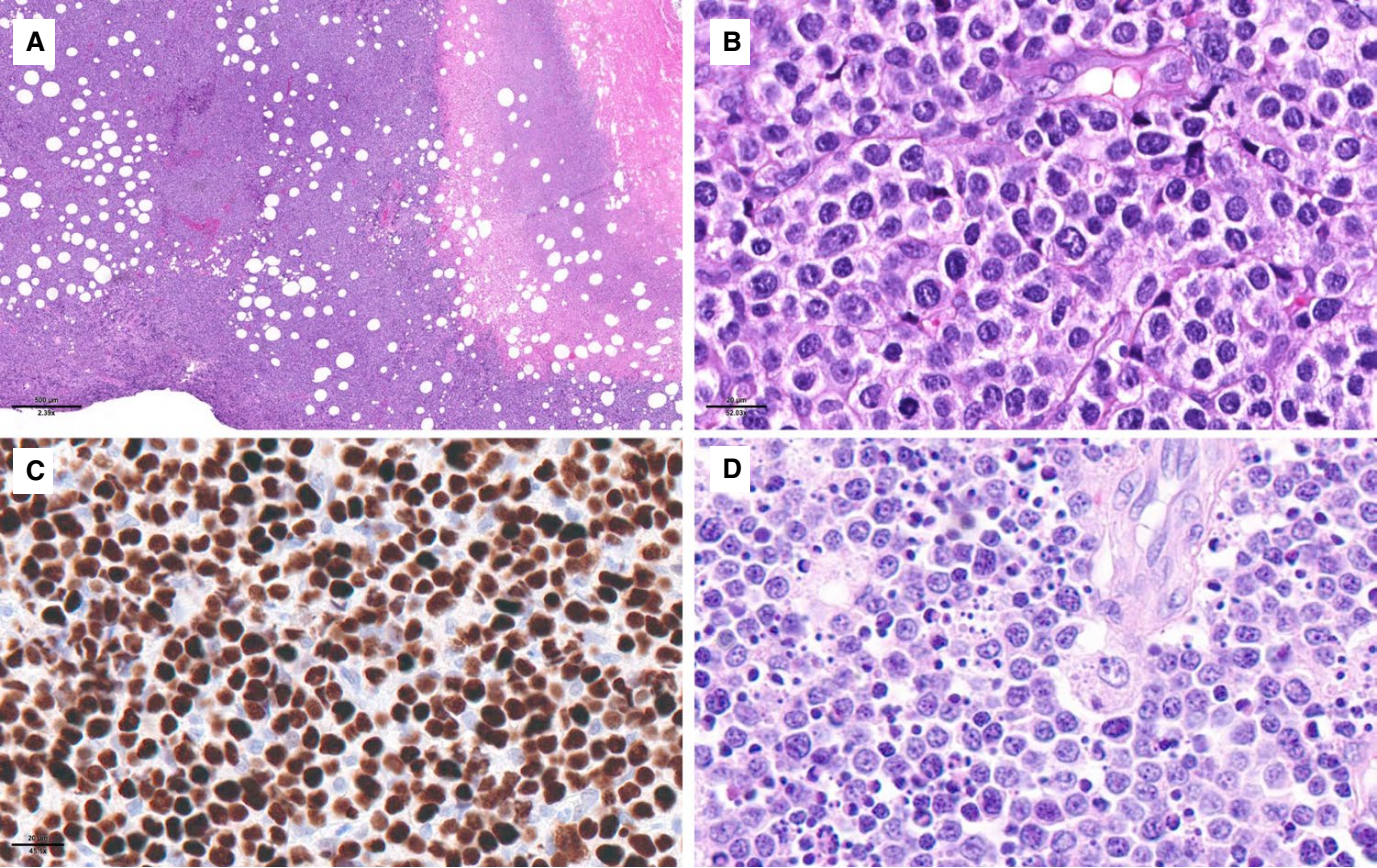
– Monomorphic epitheliotropic intestinal T-cell lymphoma with atypical features. **A**–**C** atypical MEITL with **A** large areas of necrosis distant from the surface ulcer, **B** pleomorphic medium to large cell cytomorphology, and **C** strong p53 expression (and *TP53* mutation by genomic profiling); **D** atypical MEITL consisting of cells with a blastic appearance, with abundant apoptosis and a starry sky pattern

While most cases consist of monotonous, small to medium-sized lymphoid cells with dispersed chromatin and inconspicuous nucleoli (Fig. 4), a minority shows cellular pleomorphism, larger cell size, and vesicular chromatin and/or prominent nucleoli, or may have a high-grade morphological pattern with abundant apoptosis and a starry-sky appearance (Fig. 5) [23, 70]. Some cases have necrosis distant from the ulcerated surface and invasion of vessel walls [70]. Unlike EATL, there is usually no prominent inflammatory infiltrate. The peritumoral mucosa shows prominent epitheliotropism and lesser involvement of the submucosa and muscularis propria [8]. The distant mucosa may contain patchy foci with increased IELs without villous atrophy [8].

By immunohistochemistry [8, 16, 23, 61, 64, 68, 70], MEITL cells are typically CD8 + and CD56 + (rarely one is missing), CD2 + , CD3 + , CD4 − , CD5 − , CD7 + , and CD30 − . Cases with atypical morphology have a similar immunophenotype [23, 70]. Most cases are TIA1 + ; expression of granzyme B or perforin is variable, but most cases have an activated cytotoxic phenotype. Aberrant expression of CD20 or other B-cell antigens is seen in about 20% of cases. TCR is usually expressed, more commonly TCRγδ than TCRαβ; some cases are TCR-silent or rarely coexpress both TCR isoforms. Expression of MATK is characteristic [64, 65]. Approximately one-third of cases express MYC, partly reflecting *MYC* gene locus alterations [70]. One study identified spleen tyrosine kinase (SYK) as a distinctive marker for MEITL (95% vs 0% in EATL), pointing to a role of enhanced TCR signalling in MEITL [44]. EBV may be seen in scattered B cells but is consistently absent in the neoplastic cells.

#### Molecular and genetic features

Most cases show monoclonal rearrangements of TCR genes. Copy number alterations in common with EATL include gains of 9q, 1q, and 7q [17, 40, 66]. Loss or gain of 8q is significantly more frequent in MEITL than EATL [17, 64] and *MYC* gene locus alterations are present in 20% [70]. Most cases harbor disruptive mutations or deletions of *SETD2*, which encodes a lysine methyltransferase, resulting in reduced or absent trimethylation of lysine 36 in histone H3 (H3K36me3) [52, 70]. Most cases also harbour mutations in JAK-STAT pathway genes or their negative regulators, most commonly gain-of-function mutations in *STAT5B* (> 60%) and/or *JAK3* (35–50%) [23, 45, 46, 52, 70]. Mutually exclusive alterations affecting the MAPK pathway (*BRAF, KRAS, NRAS*) are observed more frequently than in EATL, collectively in about 80% of cases [46, 52]. Mutations of *GNAI2*, encoding a subunit of guanine nucleotide binding proteins, have been reported in 9–21% [23, 45, 70]. Mutations in epigenetic regulators other than *SETD2*, including *CREBBP* histone acetyltransferase, *EP300*, *EZH2,* and *ARID1*, are infrequent [52].

#### Differential diagnosis

The morphology and immunophenotype of MEITL are distinct from those of EATL (Table 3). Although increased IELs may be seen distant from the tumor in MEITL, villous atrophy is generally absent. Expression of CD56 may suggest a diagnosis of ENKTCL, but MEITL is EBV-negative. Expression of B-cell markers in some cases may lead to considering a B-cell lymphoma.

### Intestinal T-cell lymphoma, NOS

ITCL, NOS [1] is a diagnosis of exclusion reserved for primary gastrointestinal lymphomas that do not qualify for EATL or MEITL after exclusion of intestinal involvement by another T-cell lymphoma like ENKTCL, ALCL, or HTLV-1-associated adult T-cell lymphoma/leukemia. This designation can also be used when the quality of a biopsy is insufficient (too small, inadequate sampling) to properly assess the features necessary to address the differential diagnosis.

**Fig. 6:**
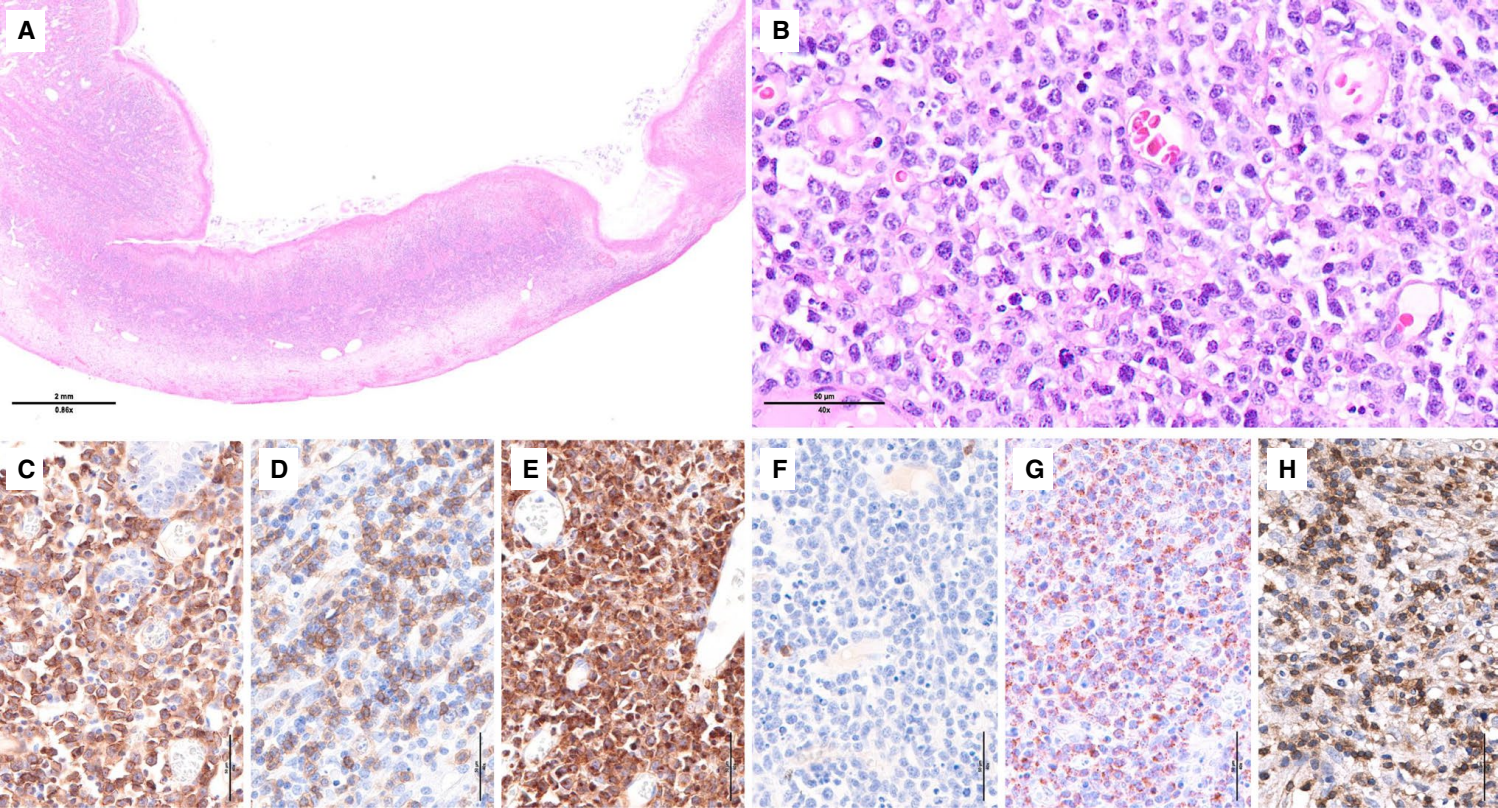
- Intestinal T-cell lymphoma, NOS. **A** Panoramic view of a small bowel section showing a transmural tumor with perforation; **B** at high magnification the tumor is composed of large pleomorphic cells and numerous mitoses are seen; **C**–**H** by immunohistochemistry the neoplastic cells are **C** CD4 + , **D** CD5 + / − , **E** CD7 + , **F** CD8 − , **G** TIA1 + , and **H** TCRbetaF1 + / −

This group of tumors, which represents a small fraction of ITCLs [78], is not well characterized and likely heterogeneous [23, 29, 61]. ITCL, NOS seems to more commonly affect the colon or the stomach than the small intestine and ~ 50% of the patients have stage III/IV disease at the time of diagnosis. Most ITCL, NOS are CD4 − /CD8 − or CD4 + and express TCRβF1 (Fig. 6). They may express cytotoxic molecules and CD30. Some are positive for EBV. Genetic data are scarce, but mutations in genes overlapping with those altered in MEITL and EATL have been reported (*JAK3*, *JAK1*, *STAT5B*, *DNMT3A*, *SETD2*) [23, 29, 46].

### Indolent clonal T-cell lymphoproliferative disorder of the gastrointestinal tract

Indolent T-cell LPD of the gastrointestinal tract was introduced as a provisional entity in the 2017 WHO classification [1] and is upgraded to a definite entity in the 2022 ICC, with addition of the term “clonal” to reinforce the importance of determining clonality for the diagnosis and distinguishing it from non-clonal reactive conditions.

Mucosal involvement is frequently multifocal with a predilection for the small bowel and colon rather than the stomach. Mesenteric lymph nodes may be enlarged. Symptoms include diarrhea, vomiting, bloating, and abdominal pain. Some patients have been misdiagnosed as RCD; others have a history of inflammatory bowel disease, autoimmunity, or infection [35, 50, 60]. The disease usually follows a chronic, indolent course. Most patients are alive with persistent disease after several years of follow-up and occasional complete remissions have been reported. However, isolated cases of spread to liver, lymph nodes, tonsils, bone marrow, and peripheral blood have been reported, and death from disease progression has been reported at 10–27 years after diagnosis in several patients [7, 35, 37, 50, 56, 60]. Hence, the disease has also been referred to as “indolent intestinal T-cell lymphoma” by some authors [37].

#### Morphology and immunophenotype

**Fig. 7:**
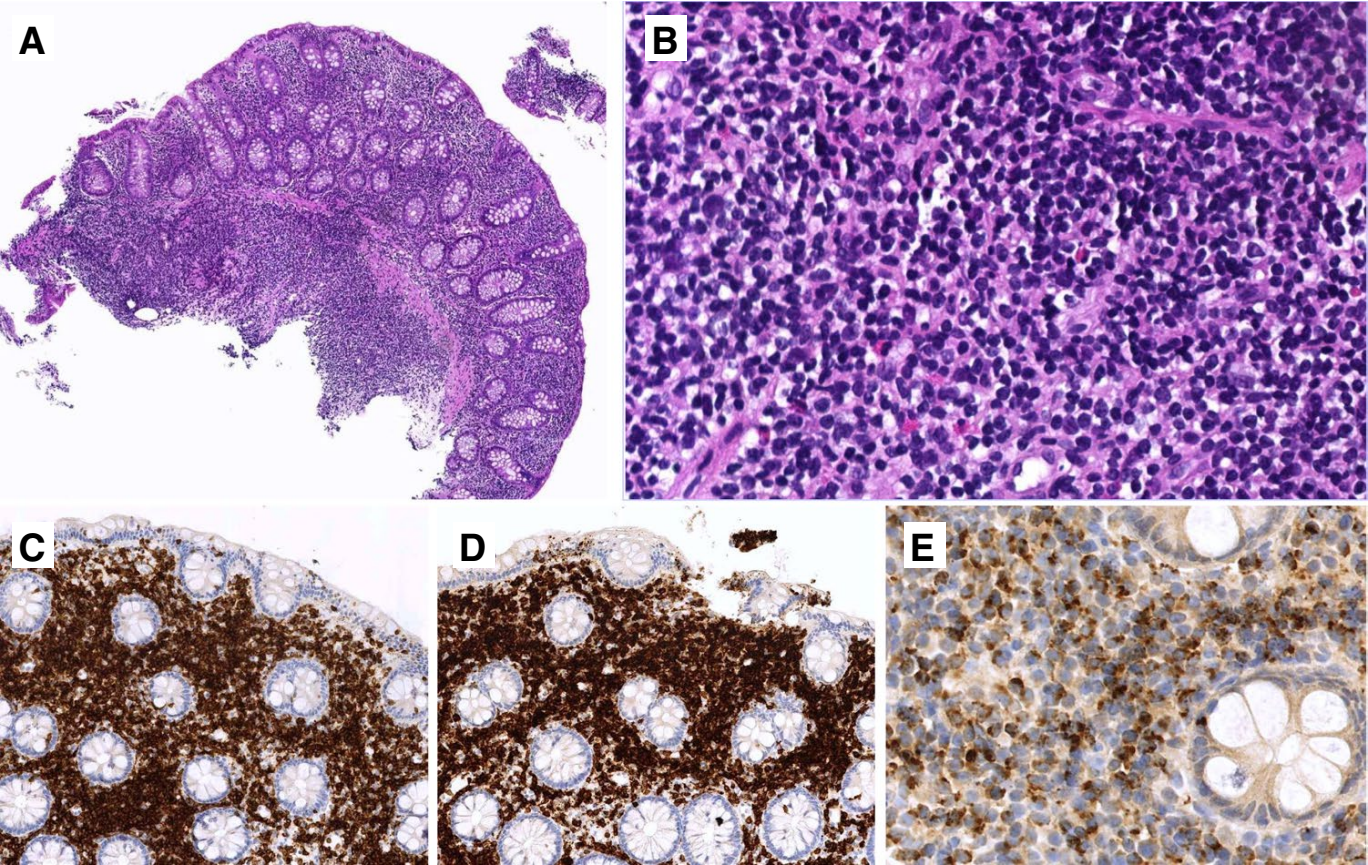
- Indolent clonal T-cell lymphoproliferative disorder of the gastrointestinal tract. **A** Colonic endoscopic biopsies showing a diffuse and dense basophilic infiltrate in the mucosa and submucosa; **B** the infiltrate consisted of small, bland lymphoid cells; **C**–**E** by immunohistochemistry the lymphoid cells are **C** CD3 + , **D** CD8 + , and **E** TIA1 + . Courtesy of Prof. Christiane Copie-Bergman

Endoscopy may show nodules, mucosal polyps, fissures, and erosions. Histologically (Fig. 7), the mucosa contains a non-destructive proliferation of small, monomorphic lymphocytes, which may infiltrate the muscularis mucosae or submucosa. Mitoses and apoptosis are inconspicuous; vascular invasion and necrosis are absent. Intestinal crypts are hyperplastic but villous atrophy is infrequent, although IELs may be encountered. Scattered granulomas, lymphoid follicles, or admixed eosinophils may be seen. Transformed cases have atypical cells and resemble PTCL.

Indolent clonal T-cell LPD of the gastrointestinal tract is CD3 + , CD2 + , and TCRβF1 + [50, 56, 58] Some cases show downregulation or loss of CD5 and/or CD7. Most cases are CD4 + , less frequently CD8 + , or sometimes double-negative (CD4 − /CD8 −) or double-positive (CD4 + /CD8 +). They lack T-follicular helper-cell markers, with variable expression of t-bet and GATA-3 [60]. CD8 + cases express TIA1. CD103 may be expressed; CD56 is usually negative and there is no association with EBV. Ki67 proliferation index is very low (< 5%). In one case, aberrant expression of CD20 was observed. Transformed cases may express MUM1/IRF4, CD30, CD25, perforin, and granzyme B.

#### Molecular and genetic features

Genetic data are scarce but suggest a different pathogenesis for CD4 + and CD8 + cases [50, 56, 60]. A recurrent *STAT3::JAK2* fusion has been found in a subset of CD4 + cases [56, 60]. Cases harboring the *STAT3::JAK2* fusion show phosphorylation of STAT5 and might be sensitive to JAK-inhibitors [56, 57]. *STAT3* mutations and *SOCS1* deletion represent other recurrent alterations found mainly in CD4 + cases [50, 60]. Other aberrations include loss-of-function mutations in epigenetic modifier genes (*TET2*, *DNMT3A*, *KMT2D*) and structural alterations of the *IL2* gene in CD8 + cases [60].

#### Differential diagnosis

Indolent clonal T-cell LPD of the gastrointestinal tract should be distinguished from both inflammatory disorders and intestinal lymphomas. Clues to the correct diagnosis include the superficial nature of the lesions, absence of mass formation and/or destructive growth pattern, bland cytology, clonal TCR gene rearrangements, and low proliferation index.

### Indolent NK-cell lymphoproliferative disorder of the gastrointestinal tract

Indolent NK-cell LPD of the gastrointestinal tract is a new term encompassing cases that have been called NK-cell enteropathy or lymphomatoid gastropathy [36, 63]. The finding of somatic mutations in a subset these cases supports its neoplastic nature and inclusion in the classification.

Less than 50 cases have been documented (reviewed in [14]) Most patients were asymptomatic or presented with mild non-specific gastrointestinal symptoms. The majority present in the stomach or duodenum, in several upper gastrointestinal sites, or involve both the upper and lower gastrointestinal mucosa. Several gastric cases had concomitant *H. pylori* infection. The lesions may spontaneously resolve or persist despite various therapies for up to several years in some patients. There are no reports of progression to aggressive disease. Interestingly, however, similar lesions have been reported involving abdominal lymph nodes, vagina, and gallbladder.

#### Morphology and immunophenotype

**Fig. 8:**
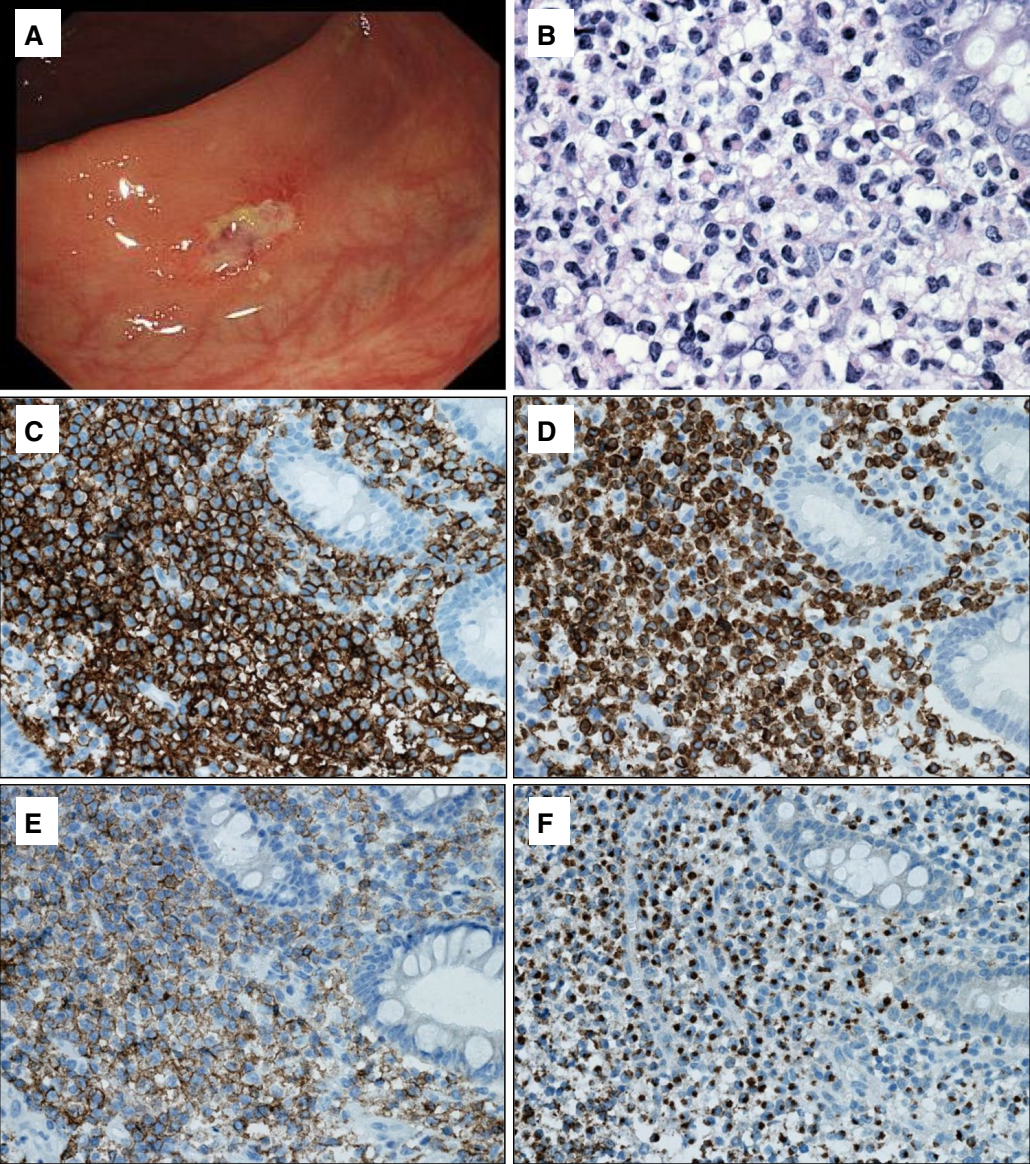
- Indolent NK-cell lymphoproliferative disorder of the gastrointestinal tract. **A** Endoscopic view of the stomach, showing an ulcerated lesion; **B** at high magnification the mucosa contains an infiltrate of medium-sized atypical lymphoid cells; **C**–**F** by immunohistochemistry, the cells are **C** CD3 + , **D** CD7 + , **E** partially CD8 + , and **F** TIA1 + . EBV was negative (not shown)

Endoscopically, the mucosa shows erythema, flat or polypoid lesions, superficial erosions, or ulcers (Fig. 8A). Histologically (Fig. 8B–F), the lamina propria is expanded by an atypical diffuse infiltrate of medium to large lymphoid cells with clear or slightly eosinophilic cytoplasm, fine chromatin, and inconspicuous nucleoli. The infiltrate may show epitheliotropism and produce glandular destruction, but lacks angiocentricity and necrosis. The atypical cells feature a CD2 + / − , cCD3 + , CD4 − , CD5 − , CD7 + , CD8 − / + , CD56 + NK-cell immunophenotype with an activated cytotoxic profile, and are EBV-negative. The Ki67 proliferation index is variable.

#### Molecular and genetic features

All cases lack clonal TCR rearrangements. A recurrent somatic *JAK3* mutation consisting of a small in-frame deletion in exon 12 was identified in 3/10 cases in one study and may suggest potential therapeutic targets in some patients [75]. Non-recurrent mutations involving *PTPRS*, *AURKB*, *AXL*, *ERBB4*, *IGF1R*, *PIK3CB*, *CUL3*, *CHEK2*, *RUNX1T1*, *CIC*, *SMARCB1*, and *SETD5* were found in other cases.

## Hepatosplenic T-cell lymphoma

HSTCL (reviewed in [51, 77]) is very rare, representing only 1–2% of PTCLs. HSTCL has unique clinicopathologic features, especially the distribution of the neoplastic cells in the sinusoids of the spleen, bone marrow, and liver, and the lack of lymph node involvement. Most cases are derived from γδ T cells and a smaller subset from αβ T cells. They have a non-activated cytotoxic phenotype.

Most patients are young adults (median age, 35 years), more often males. Up to 40% of cases occur in the setting of chronic immune suppression, particularly in solid-organ transplant recipients or patients with autoimmune disorders, most often Crohn’s disease treated with thiopurines or anti-TNF agents. HSTCL also has been reported following hematological neoplasms and in patients with *Plasmodium falciparum* infection. Patients usually present with splenomegaly and hepatomegaly without peripheral lymphadenopathy, and with B symptoms and cytopenias, especially thrombocytopenia. An overt leukemic phase is rare at presentation but may occur later in the disease. The disease is resistant to anthracycline-based chemotherapy and most patients succumb to their disease within two years of diagnosis.

### Morphology and immunophenotype

**Fig. 9:**
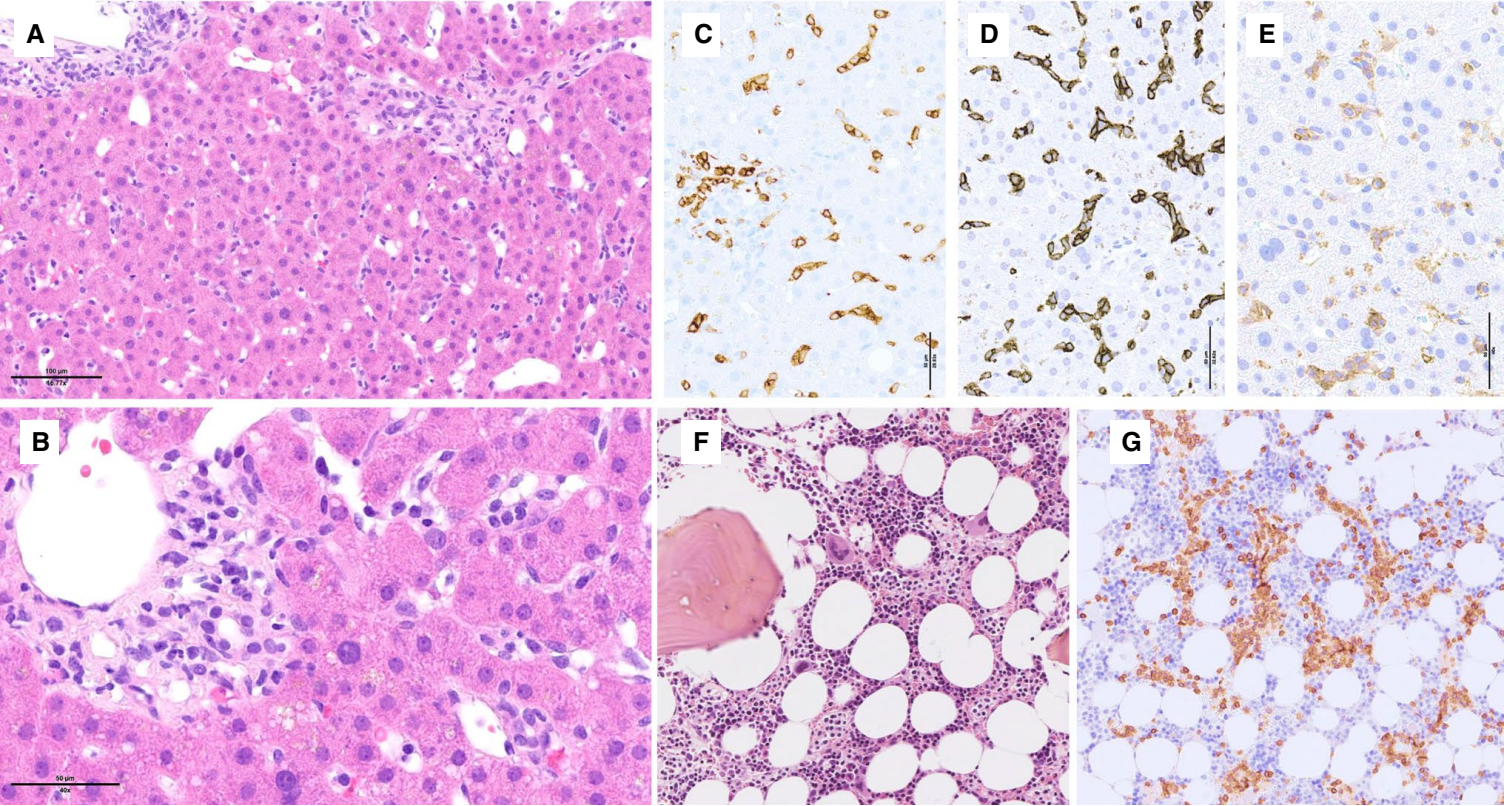
- Hepatosplenic T-cell lymphoma. **A**–**E** This liver biopsy involved by HSTCL shows **A**–**B** a mild infiltrate of mildly atypical lymphoid cells scattered or in small aggregates in the sinusoids and portal tracts; by immunohistochemistry, **C**–**E** the cells are positive for **C** CD8, **D** TCR delta, and **E** CD56. **F**–**G** This bone marrow biopsy involved by HSTCL shows **F** increased cellularity and **G** CD3 immunostaining highlights numerous T cells in a sinusoidal distribution

In the spleen, HSTCL diffusely involves the sinuses and to a variable extent the cords of the red pulp, while the white pulp is atrophic. HSTCL cells are usually medium-sized, with round or slightly irregular nuclei, condensed chromatin, and inconspicuous nucleoli. The cytoplasm is pale without granules. Cases with smaller or larger/pleomorphic cells or blastoid morphology have been reported, especially during progression. Liver infiltration shows a sinusoidal pattern with or without portal aggregates (Fig. 9A–E). The bone marrow is almost constantly involved (Fig. 9F–G), and most diagnoses are made on bone marrow samples since splenectomies are nowadays rarely performed. Infiltration of the bone marrow sinuses is highly characteristic, though often subtle and best highlighted by immunohistochemistry, and therefore an important diagnostic criterion. The myeloid component of the marrow is generally hypercellular and can show slightly dysplastic features.

HSTCL cells are CD2 + , CD3 + , CD5 − , CD7 + / − , CD30 − , CD56 + / − , and CD4 − /CD8 − (rarely CD4 − /CD8 +), usually with a non-activated cytotoxic phenotype (TIA1 + and negative for GzB and perforin). Most cases are derived from γδ cells, but up to 25% are Tαβ cells and < 5% of the cases show a TCR-silent phenotype at presentation or relapse. The gene expression profile of HSTCL unifies γδ and αβ cases, and encompasses overexpression of NK-related genes encoding KIRs and high expression of sphingosine-1-phosphatase receptor 5 (S1PR5) involved in cell trafficking [67]. Overexpression of SYK with sensitivity of HSTCL cells to SYK inhibitors has also been emphasized.

#### Molecular and genetic features

The most frequent genetic aberration in HSTCL is isochromosome 7q (80% of cases), which frequently co-occurs with trisomy 8 (50%), irrespective of αβ/γδ lineage [2, 74]. Although not specific to HSTCL, these abnormalities support the diagnosis. Chromosome 7 imbalances have been linked to overexpression of *ABCB1*, *RUNDC3*, *PPP1R9A*, and *CHN2* genes, which may be responsible for the intrinsic drug resistance of HSTCL cells [21].

The genomic landscape of HSTCL [38, 47] comprises loss of-function mutations in *SETD2* and mutations in other chromatin-modifying genes (*INO80*, *TET3, SMARCA2)* in 62% of cases. Mutations in signaling genes such as *STAT5B*, *STAT3*, and *PIK3CD* are the second most common type of alteration and a few cases harbor mutations in *TP53*, *URB5*, or *IDH2*.

#### Differential diagnosis

The main differential diagnoses in both spleen and bone marrow (Table 4) is with T-cell large granular lymphocytic leukemia (T-LGLL), a chronic indolent LPD with distinct clinical and laboratory manifestations[76], and with aggressive NK-cell leukemia (ANKCL), a disorder involving the spleen, liver, and bone marrow and also presenting in young patients with a fulminant clinical course. Distinctive features of ANKCL are the association with EBV in most cases, the NK-cell derivation, and the usually leukemic picture. In bone marrow biopsies, ANKCL cells infiltrate in a diffuse interstitial pattern.

**Table 4:** Differential diagnosis between hepatosplenic T-cell lymphoma (HSTCL), T-cell large granular lymphocytic leukemia (T-LGLL) and aggressive NK-cell leukemia (ANKCL)

	HSTCL	T-LGLL	ANKCL
Epidemiology	Mostly young adults (median age 35y), M > F, immunosuppression	Older adults (median age > 60y), M = F	Adults, more common in Asia and Latin America
Presenting features	B symptoms, cytopenias, marked splenomegaly, no lymphadenopathy	Neutropenia, variable lymphocytosis, splenomegaly, autoimmune manifestations, no lymphadenopathy	B symptoms, variable leukemic spread, cytopenias, hemophagocytic syndrome, lymphadenopathy, and splenomegaly may be present
Clinical course	Aggressive	Indolent	Aggressive, fulminant or subacute
Organs involved	Spleen, liver, bone marrow	Spleen, bone marrow, peripheral blood, liver	Bone marrow, peripheral blood, spleen, other organs (liver, lymph nodes, skin…)
Spleen	Red pulp involvement, often atrophic white pulp	Red pulp involvement, often hyperplastic white pulp	Red pulp (subtle), wall vessels
Bone marrow	Hypercellular, selective intrasinusal infiltration	Hypo- to hypercellular, sinusal, and interstitial infiltration, B cell nodules, + / − maturation arrest	Interstitial and diffuse, subtle to dense lymphoid infiltrate; hemophagocytosis common
Cytology	Medium-sized monomorphic cells with sometimes hairy projections	Small lymphocytes with minimal atypia, ample cytoplasm, azurophilic granules	Medium to large pleomorphic lymphocytes, azurophilic granules
EBV	Negative	Negative	Positive
Immunophenotype	CD3 + CD4 − CD5 − CD8 − / + CD56 + / − CD57 − , TIA1 + , TCRγδ > αβ	CD3 + CD4 − / + CD5 + CD7 − / + CD8 + CD56 − CD57 + , TIA1 + perforin + /GzB + , TCRαβ > γδ	sCD3 − cCD3 + CD4 − CD5 − CD8 − CD16 + CD56 + CD57 − TIA1 + , perforin + /GzB + , TCR −
Genetics	Iso 7q; mutations: *STAT5B* (35%), *STAT3* (rare), epigenetics (*SETD2*, *INO80*, *TET3*, *SMARCA2*)	Mutations: *STAT3* (30–40%), *STAT5B* (rare)	Del(6q), del(11q); mutations: *STAT3*(20%), *TP53* (30–35%), others (*DDX3X*, *BCOR*, *TET2)*

## Breast implant-associated anaplastic large cell lymphoma

BIA-ALCL is defined clinically by its vicinity to a breast implant and otherwise resembles systemic ALK-negative ALCL morphologically and immunophenotypically. It was a provisional category in the 2017 WHO classification and is upgraded to a definite entity in the 2022 ICC based on its distinctive genomic and molecular features [15, 18, 31, 49].

BIA-ALCL occurs in women (or rarely men) with textured prostheses implanted for cosmetic or reconstructive purpose after a median time interval of 8–11 years [12]. Most patients present with a periprosthetic effusion and are cured by capsulectomy alone. A minority of cases present as an infiltrative tumor mass, with or without an effusion, or uncommonly with regional lymphadenopathy, which both represent adverse prognostic factors [20, 27].

### Morphology and immunophenotype

**Fig. 10:**
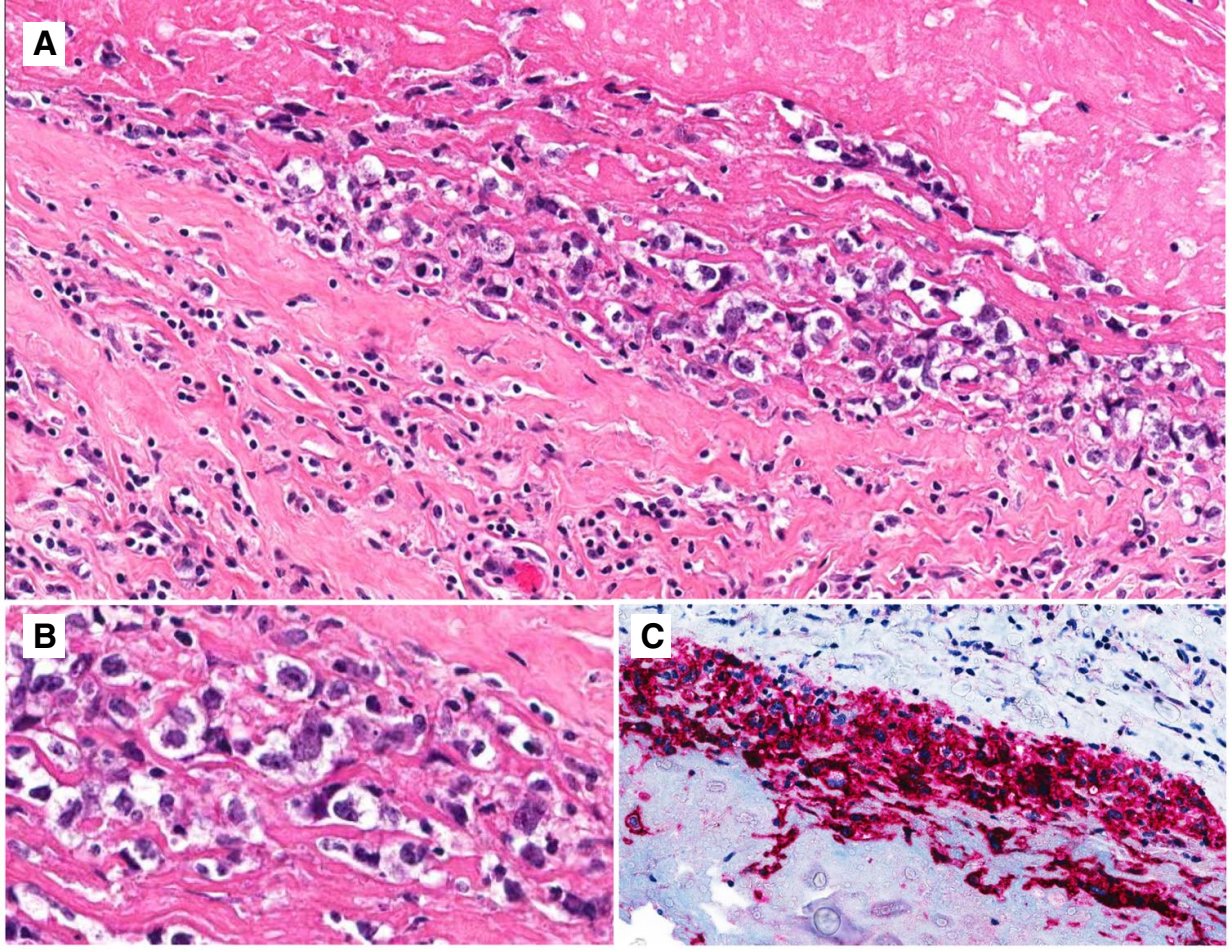
-Breast implant-associated anaplastic large cell lymphoma. **A** Low magnification of the surface of the capsule showing large atypical cells embedded in fibrin; **B** closer view of the large anaplastic cells; **C** strong CD30 expression

BIA-ALCL cells are large, with irregular nuclei, prominent nucleoli, and abundant, sometimes vacuolated cytoplasm. Capsulectomy specimens contain large anaplastic cells embedded within proteinaceous and fibrinous material at the capsular surface (Fig. 10), and may show varying degrees of capsular infiltration. Infiltration into the breast may be associated with pronounced inflammation, eosinophilia, necrosis, or sclerosis [27]. Lymph node involvement is often partial and can feature a sinusoidal, perifollicular, diffuse, or Hodgkin-like pattern, often with fibrosis [20].

BIA-ALCL cells are by definition strongly and diffusely CD30 + and ALK − . Most cases express CD2, CD4, CD43, CD45, and EMA; CD3 and CD15 are expressed in < 50% of cases and CD7 and CD5 are usually negative. A minority is CD8 +. Approximately two-thirds have an activated cytotoxic phenotype. FOXP3, CD25, and PD-L1 expression have been reported. EBV is negative.

In cases of suspected malignant peri-implant effusions, best-practice guidelines recommend obtaining a minimum of 10–50 ml of fluid for smears, cell block preparation, and immunohistochemical and molecular studies. Capsulectomy specimens should be sampled extensively for evaluation of capsular infiltration and margins, and the use of a tumor-node-metastasis (TNM) staging system is recommended, given that further therapy is guided by capsular infiltration and completeness of resection [11, 26].

### Molecular and genetic features

BIA-ALCL carry monoclonal TCR gene rearrangements and demonstration of clonality may be useful in supporting the diagnosis, especially in effusions. BIA-ALCL lacks rearrangements of *ALK*, *DUSP22*, and *TP63.* Various copy number alterations have been reported, notably characteristic chromosome 20 loss [31] and *PDL1* alterations in a subset of the cases [62]. The genomic landscape of BIA-ALCL [28, 30] encompasses JAK/STAT activating mutations (most commonly *STAT3* or *JAK1*, uncommonly *STAT5B*, *SOCS3*, *SOCS1*, and *PTPN1*) and loss-of-function alterations of epigenetic modifiers, in particular *KMT2C* and *KMT2D* encoding H3K4 methyltransferases but also *CHD2*, *CREBBP*, *TET2*, and *DNMT3A*.

The gene expression profile of BIA-ALCL is distinct from that of other PTCLs and features a hypoxic signature [18, 49].

### Differential diagnosis

Most effusions occurring in the context of breast implants are reactive. Cells variably positive for CD30 are often present in reactive effusions but are usually rare and lack atypia.

Rare indolent EBV-positive large B-cell lymphomas have been described in association with BI, thought to represent a peculiar form of chronic inflammation-associated or fibrin-associated DLBCLs. These cases have large neoplastic cells that may resemble those of BIA-ALCL and commonly express CD30, but are of B-cell lineage and are EBV-positive [39, 53].

Systemic ALCL in patients with breast implants may involve in the breast; therefore, staging and clinical history are critical to distinguish BIA-ALCL from breast involvement by systemic ALCL. The infiltrative forms of BIA-ALCL may resemble Hodgkin lymphoma morphologically, but Hodgkin lymphoma is rare in extranodal locations and has a different lineage and immunophenotype.

## Conclusion

The diagnosis of T-cell infiltrates in extranodal tissues relies on careful morphologic assessment complemented by immunophenotypic and molecular data. The presenting location is often an important signpost to orient the diagnosis, and pathological data have to be integrated with clinical features, age and ethnicity, and associated symptoms, for providing a final diagnosis. The recently characterized mutational landscapes show substantial overlap but also distinct alterations that represent useful additional diagnostic markers.
